# Biodiesel Production by Methanolysis of Rapeseed Oil—Influence of SiO_2_/Al_2_O_3_ Ratio in BEA Zeolite Structure on Physicochemical and Catalytic Properties of Zeolite Systems with Alkaline Earth Oxides (MgO, CaO, SrO)

**DOI:** 10.3390/ijms25073570

**Published:** 2024-03-22

**Authors:** Łukasz Szkudlarek, Karolina Chałupka-Śpiewak, Waldemar Maniukiewicz, Magdalena Nowosielska, Małgorzata Iwona Szynkowska-Jóźwik, Paweł Mierczyński

**Affiliations:** Institute of General and Ecological Chemistry, Lodz University of Technology, Zeromskiego 116, 90-924 Lodz, Poland; lukasz.szkudlarek@dokt.p.lodz.pl (Ł.S.); karolina.chalupka@p.lodz.pl (K.C.-Ś.); waldemar.maniukiewicz@p.lodz.pl (W.M.); magdalena.nowosielska@p.lodz.pl (M.N.); malgorzata.szynkowska@p.lodz.pl (M.I.S.-J.)

**Keywords:** BEA, metal oxide catalysts, transesterification reaction, MgO, CaO, SrO, zeolite catalysts, biodiesel production

## Abstract

Alkaline earth metal oxide (MgO, CaO, SrO) catalysts supported on BEA zeolite were prepared by a wet impregnation method and tested in the transesterification reaction of rapeseed oil with methanol towards the formation of biodiesel (FAMEs—fatty acid methyl esters). To assess the influence of the SiO_2_/Al_2_O_3_ ratio on the catalytic activity in the tested reaction, a BEA zeolite carrier material with different Si/Al ratios was used. The prepared catalysts were tested in the transesterification reaction at temperatures of 180 °C and 220 °C using a molar ratio of methanol/oil reagents of 9:1. The transesterification process was carried out for 2 h with the catalyst mass of 0.5 g. The oil conversion value and efficiency towards FAME formation were determined using the HPLC technique. The physicochemical properties of the catalysts were determined using the following research techniques: CO_2_-TPD, XRD, BET, FTIR, and SEM-EDS. The results of the catalytic activity showed that higher activity in the tested process was confirmed for the catalysts supported on the BEA zeolite characterized by the highest silica/alumina ratio for the reaction carried out at a temperature of 220 °C. The most active zeolite catalyst was the 10% CaO/BEA system (Si/Al = 300), which showed the highest triglyceride (TG) conversion of 90.5% and the second highest FAME yield of 94.6% in the transesterification reaction carried out at 220 °C. The high activity of this system is associated with its alkalinity, high value of the specific surface area, the size of the active phase crystallites, and its characteristic sorption properties in relation to methanol.

## 1. Introduction

The growth of population around the world caused by the development of technology and progressive industrialization results in a continuous increase in energy demand. This energy demand is fulfilled through the use of fossil fuels [1,2,3]. Energy has become indispensable nowadays [4,5]. However, fossil fuels used on a large scale are not a renewable energy source and are depleted, and their availability is limited, which is reflected in unstable fuel prices. Moreover, their combustion has a negative impact on the environment by emitting carbon, nitrogen, sulfur oxides, unburned hydrocarbons, smoke, dust, etc. into the atmosphere [6,7]. Some of these compounds have carcinogenic properties [6]. All these inconveniences lead to the search for new, renewable, and environmentally friendly alternative fuels [8,9]. Replacing fossil fuels with their alternative counterparts may contribute to solving the global energy crisis [10,11].

One of the liquid, clean alternative biofuels that can completely replace traditional fuels or can be used as a substitute for fossil fuels is biodiesel [12]. It is a renewable fuel, mostly obtained via the transesterification reaction (the reaction between triglycerides and alcohols as a result of which we obtain esters (typical methyl or ethyl esters) and glycerol) of triglycerides derived from edible (or non-edible) oils with short-chain alcohol (most common ones are methanol, ethanol, and butanol) [13]. Triglycerides are made up of three long hydrocarbon chains, which are derived from fatty acids bonded via an ester bond to a glycerol molecule. Triglycerides are mainly found in vegetable oils, as well as animal fats and plant microalgae [14]. Many different types of oil can be used as a raw material for the transesterification reaction, including rapeseed, soybean, sunflower, canola, palm, camelina, safflower, peanut, microalgae, and algae oils [15]. From a chemical point of view, biodiesel is an alkyl ester of fatty acids with a chain length that usually ranges from 14 to 22 carbon atoms [16]. Biodiesel is a biodegradable, eco-friendly, and non-toxic alternative to diesel fuel. In addition, it is a carbon neutral fuel, which means there is no accumulation of CO_2_ [17]. It is characterized by similar physicochemical properties to diesel fuel [5]. Combustion of biodiesel results in lower hydrocarbon and particulate emissions [18,19]. Other advantages of biodiesel are as follows: high flash point, high cetane number, low viscosity, and high lubricity [19,20,21].

In practice, the transesterification reaction is carried out with the presence of catalyst. Most commonly, homogeneous base catalysts, such as sodium hydroxide (NaOH), potassium hydroxide (KOH), sodium methoxide (CH_3_ONa) or potassium methoxide (CH_3_OK), are used [22]. Homogeneous alkali catalysts allow for the achievement of full oil conversion within a short time under mild reaction conditions (temperature and alcohol/oil ratio); however, the resulting biodiesel needs to be rinsed with water and/or neutralized by an acid treatment. As a result of such procedure, a large amount of waste water is generated. Moreover, such a catalyst cannot be recycled, resulting in higher production costs of biodiesel [23,24]. In addition, homogeneous base catalysts are sensitive to moisture and free fatty acids. Free fatty acids are subject to saponification reactions, which result in the formation of soaps and decrease the efficiency of the process by consuming the catalyst and triglycerides. In addition, the presence of soaps in the final product hinders the separation of the glycerol layer. Conversely, the presence of moisture promotes the saponification reaction [25,26]. Transesterification can also be catalyzed using homogeneous acidic catalysts. The most commonly used acids are sulfuric acid (H_2_SO_4_), sulfonic acid, hydrochloric acid (HCl), organic sulfonic acid, and phosphoric acid (H_3_PO_4_) [27]. Acidic catalysts are characterized by insensitivity to free fatty acids (FFAs) and moisture content; therefore, they can be used in reactions using oils with high FFA content as a source of triglycerides. The drawback of using acids as a catalyst in transesterification is the much slower reaction compared to alkali catalysts [28]. As in the case of a homogeneous base catalyst, the acids are not separable from the reaction mixture and can not be reused in the next reaction cycle. The final products of the reaction also require neutralization and purification. Additionally, both alkali and acid homogeneous catalysts are corrosive to the reactors and equipment [29].

Due to the inconveniences involved, transesterification reactions of oils, which are catalyzed by heterogeneous catalysts, are now gaining interest. The main advantage of heterogeneous catalysts is that they can be recovered and further reused while maintaining high activity and selectivity. The cost of biodiesel production is minimized and side reactions, such as saponification, can be neglected. In addition, these catalysts are characterized by non-corrosivity, durability, and easy separation from products [30]. Economic, easily and cheaply available heterogeneous systems are sought. Heterogeneous catalysts can be divided into basic and acidic catalysts. A direct advantage of heterogeneous acidic catalysts (e.g., zeolites, heteropolyacids, Amberlyst-15, H_3_PW_12_O_40_∙6H_2_O, WO_3_/ZrO_2_, and sulfonated carbon catalysts [31,32,33,34]) is the occurrence of simultaneous esterification and transesterification reactions. However, transesterification with these systems requires high loading of catalyst, high reaction temperatures, long reaction times, and high alcohol/oil molar ratios [35]. Heterogeneous basic catalysts (e.g., ZnO, CuO, zeolites, hydrotalcites, CaO, SrO, MgO, BaO, basic polymers, MgCO_3_, CaCO_3_, BaCO_3_, and SrCO_3_ [36,37,38]) exhibit high catalytic activity in reactions performed in mild conditions [39].

Heterogeneous catalysts used in the transesterification of vegetable/algal oils are mainly systems based on alkaline earth metal oxides (MgO, CaO, SrO). Heterogenous catalysts based on alkaline earth metal oxides, used as mixtures of these oxides or as catalysts supported on the surface of the carrier system, demonstrate high efficiency in the transesterification reaction. Another advantage of choosing these catalysts is the low cost of alkaline earth metal oxides [40]. Magnesium oxide (MgO) is a well-known heterogeneous catalyst used in the transesterification process with basic properties. Its wide use is due to the fact that it retains its catalytic properties under ambient conditions and even in the presence of high moisture content [41]. Calcium oxide (CaO) is non-toxic, insoluble in methanol, cheap, and widely available. Moreover, basic CaO catalysts are environmentally friendly and have a long lifespan [42,43,44]. Strontium oxide (SrO) is used as a catalyst in transesterification reactions, but it can also catalyze the oxidative coupling of methane, esterification (esterification is a process taking place between an organic acid (RCOOH) and an alcohol (ROH) resulting in the formation of an ester (RCOOR) and water) or selective propane oxidation, etc. SrO is insoluble in vegetable oils, methanol, and fatty acid methyl esters. It is characterized by a low specific surface area, but this does not affect its activity due to the fact that SrO-based catalysts are characterized by high activity. SrO contains basic centers stronger than H_−_ = 26.5 [45,46]. 

Zeolites are microporous, crystalline aluminosilicate materials that consist of cornered silicate (SiO_4_) and aluminate (AlO_4_) tetrahedra. Adjacent tetrahedra are connected by oxygen atoms. Zeolites are characterized by a regular arrangement of tetrahedra, giving a three-dimensional arrangement of cages and pores. The dimensions of the pores and cages are within the range of microporosity, namely, measuring from 3 to 20 Å [47]. Moreover, they are characterized by a large specific surface area. It is also worth emphasizing that it is possible to control the properties of zeolites by the selection of the silica/aluminum oxide ratio in their structure. Optimizing the Si/Al ratio affects the structure of the zeolite, as well as the diameter and volume of the pores [48]. Furthermore, porosity studies of microporous zeolites often confirm the presence of mesopores in these materials, as evidenced by higher average pore diameters as well as the presence of hysteresis loops in the adsorption–desorption isotherms [49]. One of the zeolites commonly used in catalytic reactions is synthetic BEA zeolite (BEA), which is distinguished by its intersecting three-dimensional structure of 12-membered ring channels and the presence of Lewis and Brønsted acid sites. Its modification by metal cations may allow for the creation of catalytic systems with appropriate properties for application in various types of reactions, including the transesterification process [50]. Other zeolites employed in the production of biodiesel include zeolite Y, ZSM-5, mordenite, FAU-type zeolite or 13X zeolite [51]. 

In this paper, we briefly presented information on the transesterification reaction and the catalysts commonly used in this process. The introduction contains information about zeolites applied as carrier materials in the transesterification process and rare earth metals used as the active phase of heterogeneous catalysts. The experimental part presents the methodology for the preparation of catalysts based on rare earth metals supported on BEA zeolite. The catalysts were obtained by a conventional wet impregnation method, and then tested in the transesterification process. In the next stage, their physicochemical properties were determined, including alkalinity, phase composition, textural properties, sorption properties in relation to methanol, and their morphology. The obtained results of physicochemical properties were correlated with their activity in the tested reaction.

In the literature data concerning heterogeneous catalysts used in biodiesel production, there is no information available regarding MgO, CaO, and SrO catalysts supported on BEA zeolite. Moreover, there are no data describing the impact of the BEA zeolite composition (Si/Al ratio) on the physicochemical and catalytic properties of the prepared catalysts in the transesterification process. The research conducted on this gap in the literature will allow for gaining knowledge and can be used to explain the mechanism of the investigated reaction carried out on CaO, MgO, and SrO catalysts supported on BEA zeolite. Therefore, the main aim of this work was to determine the influence of the ratio of SiO_2_/Al_2_O_3_ in the structure of zeolite BEA used as a carrier for prepared catalysts and to explain the influence of the type of active phase on the reactivity of prepared catalysts in the transesterification reaction of vegetable oil with methanol. In this work, alkaline earth metal oxides (MgO, CaO, SrO) were used as the active phase of supported zeolite catalytic systems. CO_2_-TPD, XRD, BET, FTIR, and SEM-EDS techniques were used to determine the physicochemical properties of the synthesized metal oxide systems.

## 2. Results and Discussion

### 2.1. Transesterification of Vegetable Oil with Methanol Reaction

Transesterification reactions of rapeseed oil with methanol in order to produce biodiesel were carried out in an autoclave with continuous stirring of the reaction mixture for 2 h at 180 °C and 220 °C over MgO, CaO, and SrO catalysts supported on BEA zeolites, having different SiO_2_/Al_2_O_3_ ratios (Si/Al = 25 and 300). MgO and CaO catalysts supported on zeolite BEA have been calcined at 500 °C in an air atmosphere for 4 h and tested in biodiesel production. While SrO/BEA catalysts were calcined at 600 °C in an air atmosphere for 4 h. The conversion of oil-derived triglycerides (TGs) and the yield of fatty acid methyl esters (FAMEs) were evaluated by the HPLC method and were used to express the catalytic activity of the investigated catalysts. High-performance gas chromatography was used to analyze the products of the transesterification reaction and determine the conversion of triglycerides due to the speed of analysis and the lack of the need to use long-lasting derivatization that consumes large amounts of reagents. HPLC analysis has drawbacks, including the difficulty in separating the obtained products and their quantitative evaluation as a result of overlapping chromatographic peaks, but we did not need to determine the quantity of each product. Therefore, we decided to analyze the product of biodiesel production process using the HPLC method [52]. The obtained results in the investigated process are presented in Table 1.

The reported triglyceride (TG) conversions and fatty acid methyl ester (FAME) yields indicate the influence of the SiO_2_/Al_2_O_3_ ratio and the alkaline earth oxide on the reactivity results of MgO, CaO, and SrO supported catalysts. Catalytic activity tests conducted at 220 °C showed that for all systems, FAME yields were higher than for transesterification reactions performed at 180 °C. On the other hand, an increase in TG conversion with increasing reaction temperature was observed for the 10% MgO/BEA (Si/Al = 25) (approximately 3% increase), 10% CaO/BEA (Si/Al = 300) (approximately 6% increase), and 10% SrO/BEA (Si/Al = 300) (approximately 16% increase) systems. For the other catalysts, similar TG conversions or lower conversions were recorded, but not by more than about 6%. Zeolite catalytic systems with alkaline earth metal oxides have activities arranged in ascending order as follows: 10% MgO/BEA < 10% SrO/BEA < 10% CaO/BEA. Magnesium oxide catalysts showed the least activity in this reaction, as observed by the lowest TG conversions and FAME. A large effect of reaction temperature on product selectivity in this reaction is evident for these catalytic materials, as FAME yields were within a large range (29.0–74.4%), while TG conversions were within a much narrower range (59.7–69.5%). Strontium oxide catalysts exhibited higher oil conversions (in the range of 71.4–87.8%) and biodiesel yields (65.4–98.5%) than the magnesium oxide counterparts. While the 10% SrO/BEA (Si/Al = 300) system showed the highest biodiesel yield (98.5%) among all the materials tested in the transesterification process. However, a large effect of the process temperature on selectivity and activity in this reaction is also seen for this system. In fact, the transesterification reaction performed over the 10% SrO/BEA catalyst at 180 °C gave much lower TG conversion and efficiency to methyl esters compared to the reaction conducted at 220 °C. Calcium oxide catalysts supported on BEA zeolite exhibited a very high conversion of oil (84.2–90.5%) with very high levels of FAME yield (64.0–94.6%). The 10% CaO/BEA (Si/Al = 25) system exhibited a similar triglyceride conversion for both lower- and higher-temperature reactions, while temperature had an effect on increasing the efficiency of biodiesel production. Calcium oxide supported on BEA zeolite featuring an Si/Al ratio of 300 gave the highest TG conversion (90.5%) with the second highest biodiesel yield (94.6%) in the transesterification reaction carried out at 220 °C. The activity results indicate the effect of the amount of silicon in the zeolite carriers and the alkaline earth metal oxide used on the efficiency of the transesterification reaction. The prepared CaO and SrO supported zeolite catalysts show high activity and selectivity, which can make them potential materials for the production of biodiesel from oils on an industrial scale.

The hydrogen forms of zeolite beta and zeolite beta modified with La^3+^ were tested in methanolysis of soybean oil by Shu et al. [50]. The pristine zeolite exhibited biodiesel yield at approx. 37% in the transesterification reaction performed at 333 K for 4 h with a methanol/soybean oil volume ratio of 0.8 and with the usage of 1 g of catalyst. CaO/ZSM-5 zeolite systems in the transesterification reaction of waste lard were investigated by Lawan et al. [53]. The catalytic tests were performed in a microwave reactor (595 Watts) at 65 °C for 1.25 h over 35 (*wt.*/*vol.*)% CaO/ZSM-5 catalyst using a catalyst amount of 8 (*wt.*/*vol.*)%. In each catalytic test, a methanol/oil ratio of 30:1 was used. Under these conditions, the biodiesel yield reached 90.89%. Luz Martínez et al. [54] investigated CaO catalysts supported on a sodium form of zeolite Y in the transesterification reaction of sunflower oil. They reported that the application of 16 wt% CaO nanoparticles catalyst in the reaction performed at 60 °C for 4 h with the methanol/oil molar ratio of 6:1 allows for the achievement of 78% methyl esters content in the final product. Authors achieved the highest FAME yield of 93.5% in the case of the process conducted for 6 h. The high catalytic activity of this system has been related to its alkaline properties. 

### 2.2. Specific Surface Area Measurements of the Catalytic Materials—Brunauer–Emmett–Teller

The specific surface area (SSA) measurements were carried out for alkaline earth metal oxide-BEA zeolite catalysts using the N_2_ adsorption–desorption method. The BET results obtained for calcined systems at 500 °C (or 600 °C for SrO-based catalysts) in an air atmosphere for 4 h are presented in Table 2. SSA results show that the 10 wt% alkaline earth metal oxide catalysts supported on BEA zeolite have a specific surface area in the range of 305 m^2^/g–404 m^2^/g. Catalytic systems introduced on BEA zeolite with an SiO_2_/Al_2_O_3_ ratio of 300 generally have a larger SSA than catalysts supported on BEA zeolite with an SiO_2_/Al_2_O_3_ ratio of 25, except for the 10% CaO/BEA (Si/Al = 300) catalyst, in which the specific surface area is about 20 m^2^/g smaller than the analogical system supported on zeolite BEA with a molar ratio of Si/Al = 25. In addition, the catalytic systems supported on zeolite with a higher silicon/aluminum ratio demonstrate larger micropore area and volume of micropores with a smaller external surface area together with pore volume and smaller average pore size (from approx. 8.27–9.85 nm measured for catalysts supported on BEA zeolite with an Si/Al ratio of 25 to 2.45–3.28 nm for catalysts supported on BEA zeolite with an Si/Al ratio of 300).

The largest specific surface area was measured for strontium oxide catalysts. The 10% SrO/BEA (Si/Al = 25) system exhibited an SSA of 401 m^2^/g, while a specific surface area of 404 m^2^/g was measured for the 10% SrO/BEA (Si/Al = 300) catalyst. It is noteworthy that among all the studied catalysts, the 10% SrO/BEA (Si/Al = 300) system exhibited the highest FAME yield (98.5%) in the transesterification reaction carried out at 220 °C with a high value of TG conversion (87.8%). In addition, it has the lowest average pore size, but the highest micropore volume compared to the rest of the investigated catalysts supported on BEA (Si/Al = 300). Calcium oxide-based catalysts supported on BEA (Si/Al = 300) zeolite showed the highest TG conversion (up to 90.5%) with high methyl ester yields (up to 94.6% for the process realized at 220 °C). In addition, this catalyst was characterized by the lower SSA of 78 m^2^/g in relation to SrO-containing counterparts. While the same system supported on BEA (Si/Al = 25) was also characterized by the lower SSA of about 55 m^2^/g compared to the 10% SrO/BEA (Si/Al = 25) catalyst. However, it should be noted that the average pore sizes are larger and the calculated micropore and external surface areas by the t-plot method are smaller for the CaO catalysts in comparison to the SrO catalysts supported on the same type of BEA zeolite. MgO supported catalysts exhibited similar pore size, pore volume, and similar external surface area calculated by the t-plot method compared to the CaO catalysts. The only observed difference was the larger average pore size (9.85 nm) recorded for the 10% MgO (Si/Al = 25) catalyst. The catalytic activity of MgO systems in the transesterification reaction was much lower in comparison to the tested CaO/zeolite systems, which confirms that the value of SSA, average of pore sizes, micropore area, pore volume, and external surface area are not the only factors that play a crucial role in the transesterification reaction.

Carrero et al. [55] studied biodiesel production in the transesterification process of microalgae oil using BEA zeolite. The authors reported that the microporous structure of zeolite makes it difficult for large lipid molecules to penetrate during the transesterification process. Additionally, the authors of the study confirmed that the pore size in the micro-mesopore range and the number and strength of acid sites play an important role in the investigated reaction. Other authors [56] reported that changing the Si/Al ratio in the zeolite structure may affect the catalytic properties in the transesterification reaction. Therefore, optimization of the Si/Al ratio in the zeolite structure in order to achieve an optimal hydrophobic balance allows for avoiding the deactivation of the zeolite by water as a by-product and affects the zeolite acidity. In our previous work [57], we also studied the SSA of MgO catalysts supported on zeolite materials (ZSM-5 and HZSM-5) characterized by various molar ratios between Si and Al (50 and 280) in the zeolite structure. The SSA measurements performed for the studied catalysts showed that the MgO catalysts with the higher Si/Al molar ratio exhibited higher surface area, lower pore volume together with a smaller average of pore radius. The highest SSA had 10% MgO/HZSM-5 (Si/Al = 280) catalyst, which also exhibited the lowest average pore radius compared to the rest of the investigated catalysts. The synthesized supported catalysts were also tested in transesterification reaction and the obtained results showed that MgO catalysts supported on ZSM-5 or HZSM-5 zeolites with higher Si/Al ratio exhibited higher yield towards FAME production during the process carried out at 220 °C. While the same tendency concerning TG conversion values was detected only for the MgO catalysts supported on HZSM-5 zeolite. It is also worth mentioning that the MgO catalyst supported on HZSM-5 with higher Si/Al ratio (280) was characterized by the lower crystallites of MgO phase on their surface compared to the 10% MgO/HZSM-5 (Si/Al = 50) catalyst. In the case of the MgO catalysts supported on ZSM-5 zeolite with the same Si/Al ratio, we did not observe an analogical tendency. It was also proven that MgO catalysts supported on ZSM-5 zeolite with an Si/Al ratio of 50 or 280 showed higher TG conversion and FAME yield in the studied reaction. 

The textural properties of zeolite-loaded CaO catalysts were studied by Yusuff et al. [8]. The CaO phase of the prepared catalysts was derived from chicken eggshell and the prepared CaO catalysts were tested in the transesterification process. The N_2_ sorption analysis of the CaO catalysts showed that the specific surface area of ZE was higher than the pure calcined waste eggshell (SE). In addition, it should be emphasized that the specific surface area of ZE/ES catalysts increased together with the increase in the amount of zeolite introduced into the composite system. This result was explained by the alignment of the aluminosilicate layer of zeolite with CaO formed during composite catalyst calcination. The value of SSA decreased for the composite catalysts calcined at higher temperatures. The catalyst 2ZE/ES calcined at 800 °C showed the SSA value of 83.6 m^2^/g, while the same system calcined at higher temperature 900 °C exhibited the SSA value of 54.8 m^2^/g. This result is explained by the collapse of 2ZE/ES structures due to the sintering process as a result of undesirable surface changes.

The textural properties of various CaO/HZSM-5 catalysts were also studied by Zhao et al. [58]. The authors reported that in the case of the support itself and the catalyst containing 5 or 10% by weight of CaO, N_2_ adsorption–desorption isotherms confirmed their microporous nature. It was also shown that the introduction of metal oxide into the zeolite structure reduces the SSA value compared to the support system, while the determined pore volumes for CaO catalysts are similar to those obtained for the support. A similar tendency in the case of the CaO/ZSM-5 supported catalysts was observed by Ngadi at al. [59].

### 2.3. Phase Composition Studies of Catalysts

**Figure 1:**
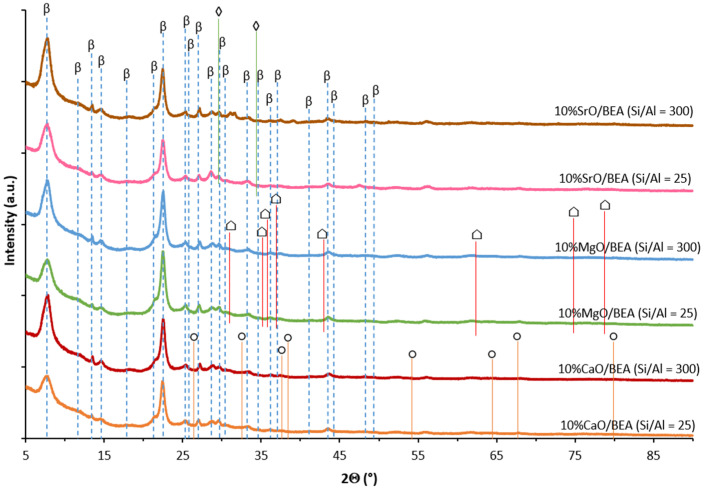
XRD patterns recorded for investigated MgO, CaO, and SrO catalysts supported on BEA zeolite with Si/Al ratio of 25 or 300 and calcined in an air atmosphere for 4 h at 500 °C (MgO, CaO catalysts) or 600 °C (SrO catalyst). (◊—SrO; ⌂—MgO; ○—CaO; β—BEA zeolite).

XRD studies have been carried out for calcined alkaline earth oxide-based catalytic materials supported on BEA zeolite to determine the phase composition of the resulting solids. XRD patterns of each particular catalytic systems are presented in Figure 1. All XRD patterns demonstrated reflexes that originated from the BEA zeolite support (2Θ = 7.5°, 11.6°, 13.5°, 14.6°, 17.9°, 21.4°, 22.4°, 25.3°, 25.9°, 27.1°, 28.7°, 29.6°, 30.4°, 33.3°, 34.7°, 36.1°, 37.1°, 41.2°, 43.5°, 44.3°, 48.4°, and 49.5°). The most characteristic XRD peaks of BEA zeolite are situated at 2Θ = 7.5° and 2Θ = 22.4°, respectively. It is worth noting that XRD patterns show higher intensity of the diffraction peak at 2Θ = 7.5° for catalysts supported on the zeolite-owned Si/Al ratio of 300. Akbar et al. [60] studied the structure and morphology of BEA zeolites and they reported that the increase in the Si/Al ratio in the zeolite structure results in the growth of the crystallites of BEA zeolites. This result also explains the higher intensity of the characteristic diffraction peaks assigned to the BEA zeolite structure located in the 2 Theta of 5–35° for the CaO, MgO, and SrO catalysts supported on BEA zeolite with higher Si/Al = 300 ratio. In addition, Akbar et al. studied the structure of Na-BEA and Li-BEA systems synthesized from NH_4_-BEA by the aqueous ion exchange technique and H-BEA by deammoniation from NH_4_-BEA zeolite. The phase composition studies of Na-BEA and Li-BEA gave evidence that the introduction of Li^+^ and Na^+^ ions into the structure of BEA zeolite via exchange ions did not change the structure of the zeolite BEA. The diffraction results showed that all systems reveal the presence of a fully crystalline structure of zeolite except for Li-BEA, in which a partial loss of crystallinity was detected.

The XRD patterns recorded for magnesium oxide catalysts show characteristic diffraction peaks attributed to the MgO at 2Θ values of 30.9°, 35.1°, 35.8°, 36.9°, 42.9°, 62.4°, 74.8°, and 78.7°. Diffractions peaks corresponding to MgO at 2Θ of 29.8°, 37.24°, 43.20°, 45.73°, 62.56°, 74.91°, and 78.83° were observed on the XRD patterns of MgO/zeolite nanostructure investigated by Jorfi et al. [49]. Park et al. [50] investigated the MgO modified zeolite and found that the XRD patterns of the material characteristic diffraction peaks are positioned at 2Θ of 37.24°, 43.20°, 45.73°, and 62.56°, which were also assigned to the MgO phase. Diffractograms of studied calcium oxide catalysts exhibit reflections that correspond to the active phase (CaO), which are located at 2Θ values of 26.4°, 32.5°, 37.6°, 38.3°, 54.2°, 64.5°, 67.7°, and 79.9°. Yusuff et al. [51] identified diffractions peaks at 2Θ of 26.6°, 32.2°, 37.3°, 53.8°, 64.1°, and 67.3° that originated from the CaO phase. Husin et al. [52] observed the XRD pattern of NiO/zeolite-CaO catalyst diffraction reflexes at 2Θ values of 29.3°, 34.1°, 38.7°, and 54.2°, which are attributed to the CaO and corresponding to (110), (111), (200), and (211) planes. While the diffraction curves recorded for strontium oxide catalysts showed diffraction peaks located at 2Θ of 29.5° and 34.3°, which are attributed to the crystallographic phase of SrO. These characteristic XRD peaks of SrO were also observed by Widiarti et al. [53] in their study of SrO catalysts supported on SiO_2_ and investigated in the transesterification reactions of soybean oil. In their paper, X-ray diffraction peaks of SrO were noticed at 2Θ values of 29.77°, 34.69°, 49.88°, and 59.33°, respectively.

Besides the phase composition analysis of the synthesized samples of alkaline earth metal oxide-zeolite BEA catalytic systems, the average crystallite size of the active phase was determined based on the Scherrer equation [61]. The diffraction patterns were processed using HighScore Plus software (ver. 3.0e, Malvern Panalytical Ltd., Royston, UK, 2012) by fitting to Pseudo–Voigt function. Determination of the crystallites size was quite difficult due to the large number of peaks originating from BEA zeolites. Table 3 presents the calculated average size of active phase crystallites. The MgO and CaO systems on BEA zeolite with an Si/Al ratio of 300 showed a smaller crystallite size than the systems impregnated on BEA zeolite characterized by an Si/Al ratio of 25. The exceptions are the systems based on strontium oxide, where for the zeolite supported catalyst with lower silicon/aluminum ratio, the average SrO crystallite size was smaller (9 nm) compared to the same system supported on the BEA zeolite with higher silicon content (Si/Al = 300), for which the calculated average SrO crystallite size was 13 nm. The 10% SrO/BEA (Si/Al = 25) system in question, as well as the 10% MgO/BEA (Si/Al = 300) catalyst, showed the smallest size of alkaline earth oxide crystallites of 9 nm. On the other hand, the largest crystallite size (16 nm) was determined for the 10% CaO/BEA (Si/Al = 25) system. Regarding the catalytic activity of the investigated systems, it can be noted that the systems with higher silicon content (Si/Al = 300) in the zeolite support resulted in higher TG conversions and biodiesel yields for the process realized at 220 °C, except for MgO-based catalysts, for which TG conversion decreased from 69.5% to 59.7%. It is also worth noting that the 10% SrO/BEA (Si/Al = 300) catalyst, for which the highest FAME yield was detected in the transesterification reaction carried out at 220 °C, had a similar crystallite size of SrO (13 nm) compared to the 10% CaO/BEA (Si/Al = 300) catalyst (14 nm), for which the highest TG conversion and second highest FAME yield was detected. The worst TG conversion value in the biodiesel production process showed MgO/BEA (Si/Al = 300) among all catalysts tested in the transesterification reaction at both temperatures. Only in the case of FAME yield values calculated for MgO/BEA (Si/Al = 300) catalyst, the yield value of fatty acid methyl esters for this system was higher than for the MgO catalyst supported on BEA (Si/Al = 25) carrier. While the obtained catalytic activity results for the investigated catalysts in the transesterification process carried out at 180 °C showed that for all catalysts TG conversion and FAME yield decreased in parallel with the increasing Si/Al ratio from 25 to 300 in catalytic material. This tendency was opposite to the average size of the oxide crystallites, except for the 10% SrO/BEA (Si/Al = 25) system, for which smaller SrO crystallites were observed on the surface of BEA zeolite. The reactivity results performed in the transesterification reaction showed that the most active system 10% CaO catalyst supported on BEA (Si/Al = 300) exhibited the highest TG conversion of 90.5% with the second highest value of FAME yield of 94.6%. This system also exhibited practically the same average size of CaO crystallites compared to the 10%CaO/BEA (Si/Al = 25) system.

### 2.4. Basic Properties of the Synthesized Catalyst Systems

In order to characterize the alkaline properties of catalytic materials, the CO_2_-TPD technique was utilized. Experiments were performed to explain the observed catalytic activity in biodiesel production of the investigated systems. Before the experiment, all studied catalysts were previously heated at 500 °C. The numbers of weak desorption centers (in the temperature range of 100–300 °C), moderate desorption centers (300–450 °C), and strong desorption centers (>450 °C) calculated from the CO_2_-TPD profiles are listed for all tested catalyst samples in Table 4. 

A weak desorption band appearing in the temperature range of 200–300 °C on the CO_2_-TPD profiles of MgO/BEA catalysts are attributed to the basic sites related to oxygen in Mg^2+^–O^2−^ pairs [62]. The desorption peak occurring at temperatures of 250–300 °C can be assigned to stronger basic centers, which are isolated O^2−^ anions [63]. Weak basic centers in CaO zeolite catalysts show desorption peaks in the temperature range of 50–210 °C, while medium strength basic centers are represented as a desorption peak on the profile in the temperature range of 220–420 °C. It is related to the presence of Ca^2+^–O^2−^ pairs [64]. Desorption of CO_2_ at temperatures approaching 600 °C is assigned to strong basic sites, which are unbound O^2−^ anions [63,65].

The highest total alkalinity was demonstrated by the 10% CaO/BEA (Si/Al = 300) system, which in catalytic activity tests (in a reaction carried out at 220 °C) exhibited the highest TG conversion among all the studied catalysts and the second highest biodiesel yield. The alkalinity of the studied systems can be ordered in descending order as follows: 10% CaO/BEA (Si/Al = 300) > 10% MgO/BEA (Si/Al = 300) > 10% CaO/BEA (Si/Al = 25) > 10% SrO/BEA (Si/Al = 25) > 10% MgO/BEA (Si/Al = 25) > 10% SrO/BEA (Si/Al = 300). The obtained results indicate the influence of the ratio of SiO_2_/Al_2_O_3_ in zeolite on catalyst alkalinity. In general, all alkaline earth oxide catalysts introduced into the BEA zeolite with an Si/Al ratio of 300 showed higher alkalinity (except for the 10% SrO/BEA system (Si/Al = 300)), which is reflected in higher oil conversions and biodiesel yields. Higher SiO_2_/Al_2_O_3_ ratio in the BEA zeolite structure is reflected in an increased number of weak and moderate strength basic centers, which explains the activity results of the systems (with the exception of strontium oxide systems, where the number of moderate strength centers was similar regardless of the Si/Al ratio in the zeolite carrier).

Meanwhile, in terms of the kind of alkaline earth metal oxides employed as the active phase in these heterogeneous systems to catalyze the transesterification reaction, the basicity is arranged in the following order: MgO/BEA < SrO/BEA < CaO/BEA for the catalysts supported on BEA zeolite with an Si/Al molar ratio of 25. While in the case of the catalysts supported on BEA zeolite with an Si/Al molar ratio of 300, the basicity forms the following sequence: SrO/BEA < MgO/BEA< CaO/BEA. In general, oxides and hydroxides of alkaline earth metals show an increase in alkalinity in the following order: Mg < Ca < Sr < Ba [66]. 

### 2.5. Morphology of the Investigated Catalysts

**Figure 2:**
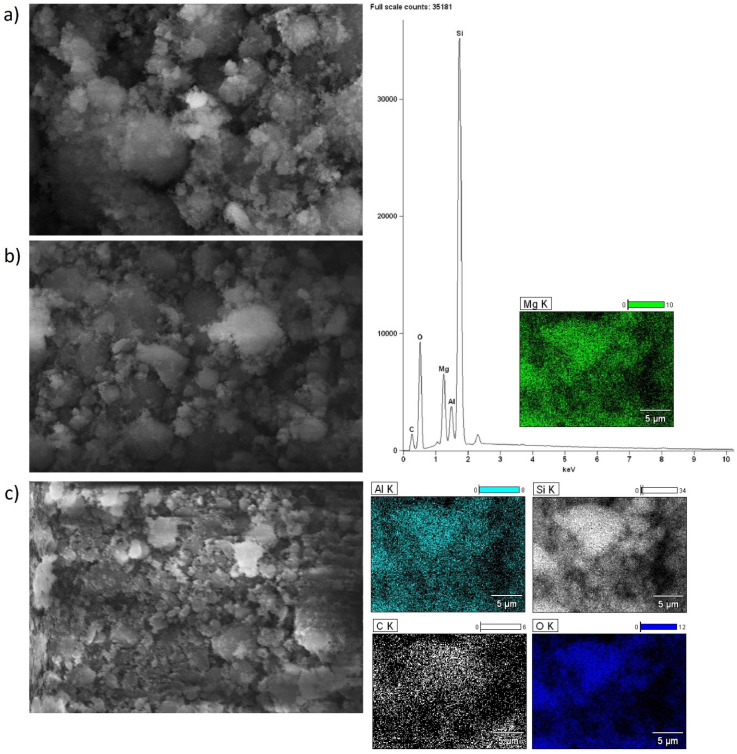
SEM-EDS images: ((**a**) magnification 5000×; (**b**) magnification 2500×; (**c**) magnification 1000×), EDS elemental mapping and EDS spectrum recorded for 10% MgO/BEA (Si/Al = 25) catalyst after calcination at 500 °C for 4 h in an air atmosphere.

**Figure 3:**
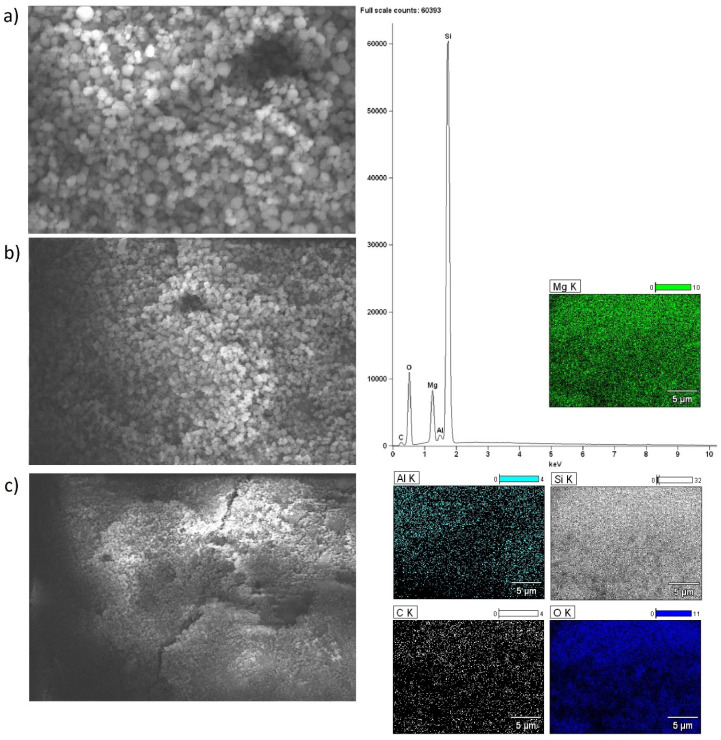
SEM-EDS images: ((**a**) magnification 5000×; (**b**) magnification 2500×; (**c**) magnification 1000×), EDS elemental mapping and EDS spectrum recorded for 10% MgO/BEA (Si/Al = 300) catalyst after calcination at 500 °C for 4 h in an air atmosphere.

**Figure 4:**
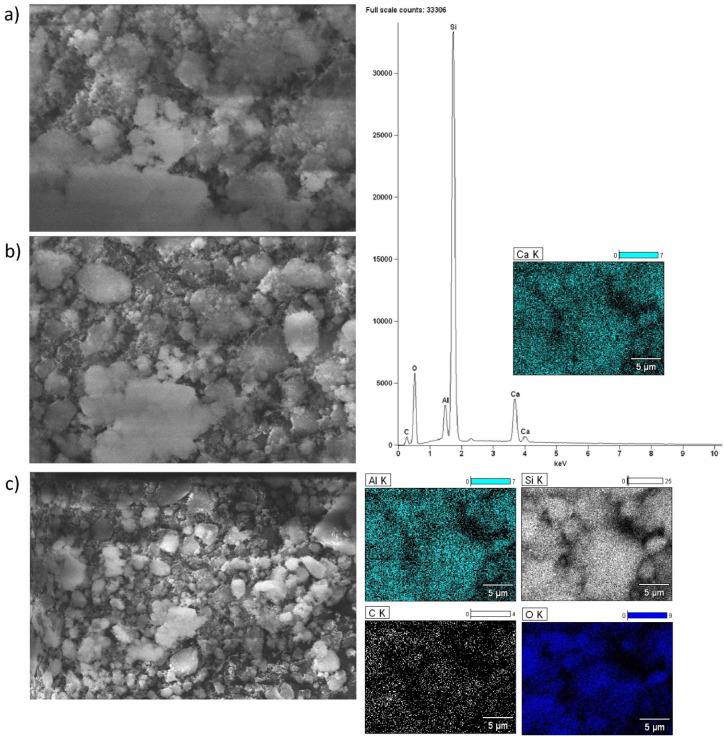
SEM-EDS images: ((**a**) magnification 5000×; (**b**) magnification 2500×; (**c**) magnification 1000×), EDS elemental mapping and EDS spectrum recorded for 10% CaO/BEA (Si/Al = 25) catalyst after calcination at 500 °C for 4 h in an air atmosphere.

**Figure 5:**
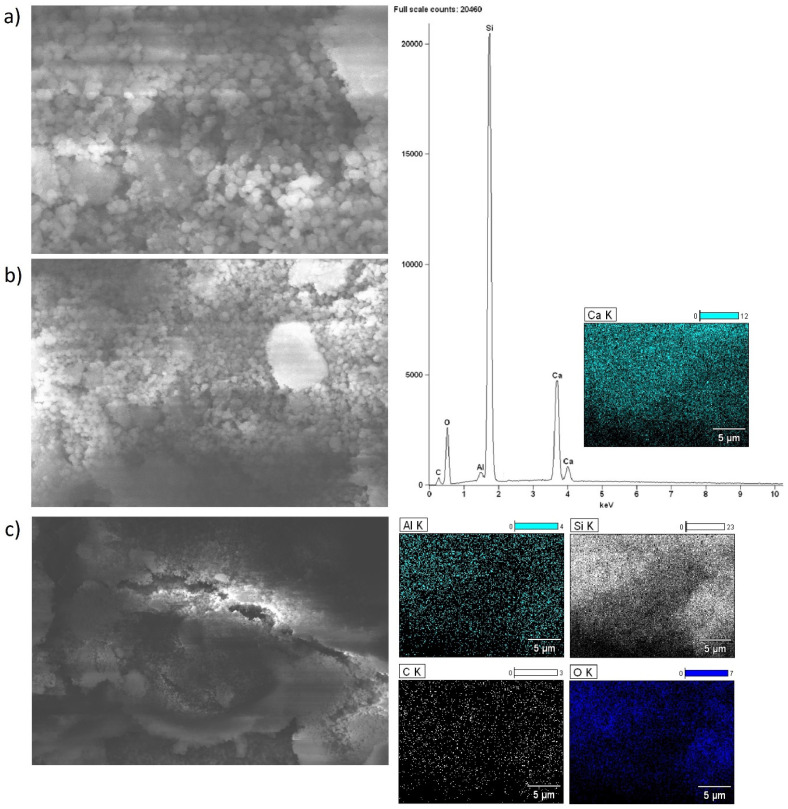
SEM-EDS images: ((**a**) magnification 5000×; (**b**) magnification 2500×; (**c**) magnification 1000×), EDS elemental mapping and EDS spectrum recorded for 10% CaO/BEA (Si/Al = 300) catalyst after calcination at 500 °C for 4 h in an air atmosphere.

**Figure 6:**
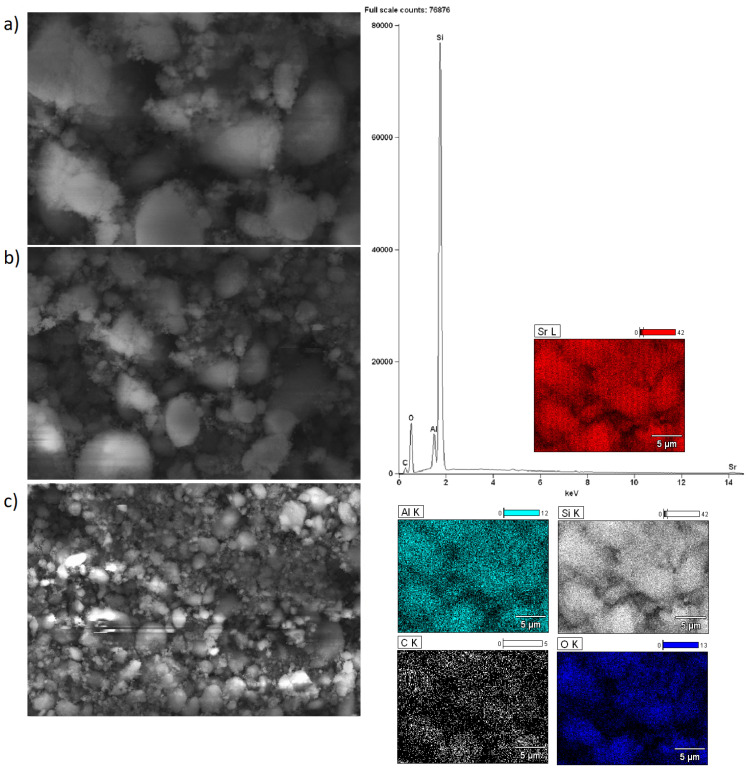
SEM-EDS images: ((**a**) magnification 5000×; (**b**) magnification 2500×; (**c**) magnification 1000×), EDS elemental mapping and EDS spectrum recorded for 10% SrO/BEA (Si/Al = 25) catalyst after calcination at 600 °C for 4 h in an air atmosphere.

**Figure 7:**
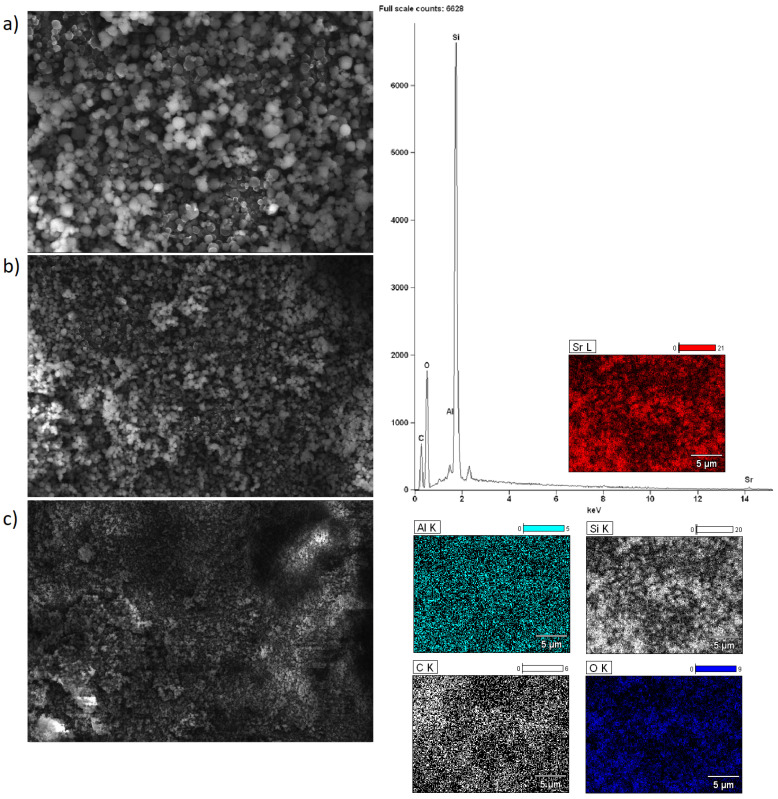
SEM-EDS images: ((**a**) magnification 5000×; (**b**) magnification 2500×; (**c**) magnification 1000×), EDS elemental mapping and EDS spectrum recorded for 10% SrO/BEA (Si/Al = 300) catalyst after calcination at 600 °C for 4 h in an air atmosphere.

Scanning electron microscopy with EDS detector was carried out for alkaline earth oxide catalysts (MgO, CaO, SrO) supported on BEA zeolite, characterized by different silicon/aluminum ratios. The purpose of the SEM-EDS measurements was to determine the elemental composition of the surface of the system calcined at 500 °C (or 600 °C for SrO/BEA catalysts) for 4 h in an air atmosphere, along with characterization of the morphology of the surface. SEM images of the catalyst surface EDS elemental maps and EDS spectra were collected for all investigated catalysts and are all shown in Figure 2, Figure 3, Figure 4, Figure 5, Figure 6 and Figure 7. Elemental composition analysis for all samples demonstrated the appearance of Si, Al, O and elements from certain alkaline earth metals (Mg, Ca, Sr) on the surface of the systems applied in oxide form as the active phase of the zeolite catalytic systems in the transesterification reaction. Figure 2 and Figure 3 present the results of SEM-EDS measurements of magnesium oxide catalysts supported on BEA zeolites. The elemental maps depict that regardless of the selected zeolite support with different Si/Al ratios, the distribution of magnesium is homogeneous. The EDS spectrum along with elemental maps and SEM images for calcium oxide catalysts on BEA zeolite with Si/Al = 25 and Si/Al = 300 ratios are shown in Figure 4 and Figure 5. Similar to MgO/BEA catalysts, calcium oxide systems also show an even distribution of calcium on the catalyst surface in the elemental map. Also, the uniform distribution of strontium on the catalyst surface is apparent in the elemental map collected for the 10% SrO/BEA catalyst sample (Si/Al = 23) and depicted in Figure 6, together with the SEM images and EDS spectrum. The elemental map collected for the 10% SrO/BEA catalytic system (Si/Al = 300) illustrates good coverage by the strontium of the inhomogeneous catalyst surface, as seen in the SEM image (Figure 7). SEM images captured for samples of catalysts supported on BEA zeolite with Si/Al = 25 ratio present an irregular grain shape, while the structure of catalyst samples supported on BEA zeolite with Si/Al = 300 ratio is quite different, as it presents a large amount of regular, close to spherical shapes of grains with small size. Summarizing the SEM-EDS measurements performed for the CaO, MgO, and SrO catalysts, a detailed analysis of the EDS results obtained for the investigated catalysts was performed, but no simple correlation was found between the content of individual metals on the catalyst surface and the reactivity results obtained in transesterification reaction (TG conversion and FAME efficiency). Based on the EDS measurements performed for the studied catalysts, the only identified correlation was the finding that the prepared catalysts, characterized by the smaller metal oxide crystallites located on the surface of BEA zeolite, showed high intensity peaks on the EDS spectra assigned to the characteristic X-ray radiation emitted from the surface of the catalyst and attributed to the Ca, Mg or Sr metals on the investigated catalysts. These measurements indicate that the size of the crystallites of metal oxide results determined using the XRD technique for the studied catalysts were confirmed by the EDS analysis.

### 2.6. Sorption Properties of Investigated Catalysts in Relation to Methanol

**Figure 8:**
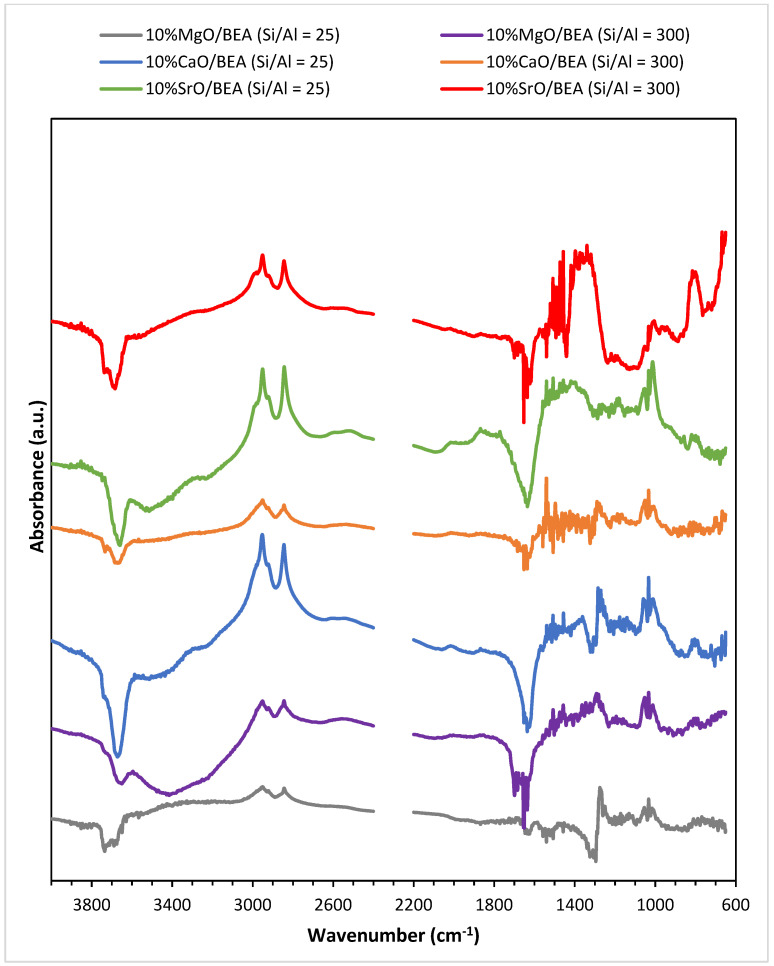
FTIR spectra of adsorbed species taken after exposure of the catalytic system (10% MgO/BEA, 10% CaO/BEA, and 10% SrO/BEA) calcined in an air atmosphere at 500 °C (or 600 °C for SrO catalysts) for 4 h, to a mixture of 5 vol% methanol—95% argon mixture at 50 °C and after the evacuation of this mixture for 10 min using argon stream. The ratio of silicon/aluminum is given in parentheses.

In order to clarify the occurring differences in the catalytic activity of alkaline earth metal oxide-containing catalysts supported on BEA zeolites, an investigation of the sorption properties of methanol was carried out using the FTIR technique. The results of FTIR studies are shown in Figure 8. There are several distinguishable characteristic IR bands readily apparent on the spectra captured for the examined catalysts. The very weak signal with the wavenumbers between 3760 cm^−1^ and 3740 cm^−1^ visible on the spectra is assigned to external silanol groups. A weak band in the region between 3617 cm^−1^ and 3569 cm^−1^ can be seen on the spectra for all investigated systems originating from the isolated Brønsted acid sites of BEA zeolite. In the IR spectra of all catalysts bands, maximum values in the range of 2958–2945 cm^−1^ are observed. This band has two additional maximas (in the range of 2933–2926 cm^−1^) for SrO catalysts and for 10% CaO/BEA (Si/Al = 25) system. This whole IR band is attributed to the (ν_asym_(CH_3_)) stretching vibrations of C-H bond [67]. Also noticeable are the bands originating from the (ν_sym_(CH_3_)) stretching vibrations in the range of 2852–2835 cm^−1^ [68,69]. Intensities of these bands were highest for zeolite catalysts containing strontium oxide as the active phase and for a system containing calcium oxide applied to a zeolite with a lower silicon/aluminum ratio. Magnesium oxide catalysts supported on BEA zeolite, on the other hand, presented the lowest intensity of these bands. The negative band appearing on all IR spectra for the catalysts studied at a wavenumber value of 1600 cm^−1^ is characteristic of modes originating from the removal of initially adsorbed water (δ(OH)) [70]. Another visible band shows that the infrared spectra for all catalysts occur in the range of 1494–1453 cm^−1^, which according to the literature comes from bending vibrations of C-H bond (δCH_3_) [67,71]. This might be related to the asymmetric (δ_asym_(HCH_2_)) and symmetric modes (δ_sym_(HCH_2_)) [67]. The intensity of this band is greater for catalysts supported on the BEA zeolite with higher Si/Al ratio. The observed band on the spectra for all catalytic systems with maxima in the range of wavenumbers from 1337 cm^−1^ to 1265 cm^−1^ refers to the δ(OH) modes of H-bonded zeolite hydroxyls [70]. Depending on the type of active phase, differences in the intensity of this band are apparent—for CaO catalysts on BEA zeolite, there was no significant change in intensity depending on the Si/Al ratio in the zeolite, while a higher intensity of this band is seen in the spectra of magnesium systems for a system on zeolite with an Si/Al ratio of 25. In contrast, a significantly more intense band for SrO/BEA catalysts is seen for zeolite with an Si/Al ratio of 300. The signals in the region of 1034–997 cm^−1^ visible on spectra for all investigated catalysts correspond to the characteristic ν(CO) stretching mode of methanol [72]. The intensity is rather the same for MgO and CaO-based zeolite catalytic systems; however, it arises for catalysts, where SrO was applied as an active phase. Correlating the FTIR results of methanol sorption with the activity results allows for explaining the lowest activity of MgO/BEA catalysts in the transesterification reaction due to the lowest intensities of the bands coming from the methanol-bound groups, indicating the weakest sorption properties. The CaO and SrO systems applied on the BEA zeolite, which exhibited better oil conversion and biodiesel yields, presented better sorption properties. The amount of adsorbed methanol on the catalyst surface is one of the first steps of the transesterification process on the catalyst surface and plays an important role during the studied reaction using the synthesized catalytic systems.

## 3. Materials and Methods

### 3.1. Preparation of the Catalytic Materials

Alkaline earth metal oxide catalysts supported on BEA zeolite were synthesized by the wet impregnation method. The ammonium form of commercial BEA zeolites, which were purchased from Zeolyst International (Kansas City, KS, USA), was used for the synthesis of the catalytic material. These zeolites were characterized by SiO_2_/Al_2_O_3_ ratios of 25 and 300, respectively. The zeolites were subjected to long-term calcination for 15 h in an air atmosphere at 500 °C in order to convert the ammonium form of the zeolites to hydrogen form. The active phase precursors were magnesium nitrate hexahydrate (Mg(NO_3_)_2_·6H_2_O), calcium nitrate tetrahydrate (Ca(NO_3_)_2_∙4H_2_O), and strontium nitrate (Sr(NO_3_)_2_) purchased from SIGMA-ALDRICH (St. Louis, MO, USA, ACS reagent, 99%). The content of MgO, CaO, and SrO in the prepared catalytic materials was 10 wt%. The studied calcium catalyst systems were then calcined in a muffle furnace in an air atmosphere at 500 °C (for SrO catalysts, the temperature of calcination was 600 °C) for 4 h.

### 3.2. Characterization of the Catalytic Material

Morphology of the surface of alkaline earth oxide systems on BEA zeolites was investigated with a HITACHI (Tokyo, Japan) S−4700 scanning electron microscope equipped with an energy dispersive spectrometer (ThermoNoran, Madison, WI, USA) (SEM-EDS). X-ray diffraction (XRD) technique was used to determine the phase composition of the prepared catalysts. A PANalytical X’PertPro MPD diffractometer in Bragg–Brentano reflectance geometry (Malvern Panalytical Ltd., Malvern, UK) with Cu Kα radiation (k = 154.05 pm) from a sealed tube in the 5–90° range of 2Θ angle was used in the study. Determination of the alkaline properties of the prepared zeolite systems was performed using the temperature-programmed desorption technique. The TPD-CO_2_ measurements were carried out using a quartz microreactor. Carbon dioxide was used as a probe-molecule during each CO_2_-TPD measurement. Before the TPD measurement, the catalyst sample was dried at 500 °C for 60 min in a helium stream (total gas flow: 40 cm^3^/min), and then CO_2_ was adsorbed onto the catalyst surface at 50 °C for 30 min. The temperature-programmed desorption of CO_2_ was carried out in the temperature range of 50–600 °C with the temperature of 25 °C, after removing carbon dioxide which was previously physiosorbed on the catalyst surface. The specific surface area of the prepared catalyst samples was determined by the nitrogen adsorption−desorption method at −195 °C using the Micrometrics ASAP 2020 analyzer. The total surface area was determined using the Brunauer–Emmett–Teller (BET) method, and the surface area and volumes of the micropores were obtained using the t-plot method. The Barrett–Joyner–Halenda (BJH) model was used to determine the average pore size. The sorption properties of the catalytic materials in relation to methanol were studied using the FTIR spectrometer (Nicolet Is50—FTIR-Thermo—Scientific, Waltham, MA, USA) equipped with a liquid–nitrogen-cooled MCT detector. A resolution of 4.0 cm^−1^ was used throughout the investigation. A total of 64 scans were taken to achieve a satisfactory signal-to-noise ratio. The background spectrum was collected at 50 °C in an argon stream. Before the measurements, argon was shifted to a mixture of 5 vol% CH_3_OH in the argon stream. The adsorption process involved exposure of the heated catalysts to 5 vol% CH_3_OH in an argon stream flowing at 40 cm^3^/min for 15 min under atmospheric pressure. After the adsorption process, the cell was evacuated for 15 min in an argon stream, and then the spectrum was collected. FTIR spectra of adsorbed species were taken after exposure of the MgO or CaO catalysts calcined in an air atmosphere at 500 °C for 4 h (or 600 °C for SrO catalysts), to a mixture of 5 vol% methanol−95% argon mixture at 50 °C and after the evacuation of this mixture for 15 min using argon stream. During all FTIR measurements, a sample weighing approximately 0.2 g was placed in a special chamber through which Ar or a 5% CH_3_OH–95% Ar mixture was passed.

### 3.3. Catalytic Activity Measurements in Transesterification of the Vegetable Oil with Methanol

All transesterification reactions were carried out in an autoclave (Parr USA, reactor volume 50 mL) equipped with a mechanical stirrer. The starting reaction mixture consists of a commercial rapeseed oil and methanol, purchased from CHEMPUR, Piekary Śląskie, Poland. The molar ratio of methanol/oil in all experiments was 9:1. Approximately, 0.5 g of catalyst was used in all catalytic tests. Before the reaction mixture, all catalysts were calcined at 500 °C (10% MgO/BEA and 10% CaO/BEA) or at 600 °C (10% SrO/BEA) for 4 h in an air atmosphere using a muffle furnace. Transesterification reactions were carried out at 180 °C and 220 °C, respectively. Reaction products were collected and analyzed using the HPLC technique (Shimadzu, Kyoto, Japan) equipped with a DAD detector (wavelength: λ = 205 nm) in order to determine triglycerides conversion and FAME yield. During the analysis of the obtained product, C-18 column and a mixture of 2-isopropanol-hexane (4/5) and methanol as eluent were used. The mobile phase gradient used during each experiment is shown in Table 5.

## 4. Conclusions

Transesterification reactions of commercial rapeseed oil with methanol, catalyzed by heterogeneous alkaline earth oxide-zeolite BEA systems, were carried out. All catalytic materials were synthesized by the wet aqueous impregnation method and tested in order to select the optimal catalyst. In addition to the catalytic tests, the physicochemical properties of the prepared catalysts were studied by CO_2_-TPD, XRD, BET, FTIR, and SEM-EDS methods to correlate these properties with reactivity results in the biodiesel production process. The obtained results confirmed the influence of the SiO_2_/Al_2_O_3_ ratio in catalysts containing alkaline earth metal oxides supported on BEA zeolite and the type of alkaline earth metal oxide as the active phase on the catalytic activity of the tested catalysts in the transesterification reaction carried out at 220 °C. The best catalytic activity results were obtained for transesterification reactions performed at 220 °C, using calcium oxide catalysts on BEA zeolites. The reactivity results showed that the reaction performed at 220 °C resulted in higher biodiesel yields over investigated catalysts compared to the reactions conducted at 180 °C. The highest oil conversion (90.5%) along with the second highest biodiesel yield (94.6%) was confirmed for the 10% CaO/BEA system (Si/Al = 300). The high activity of this system is related to the highest alkalinity among all tested catalytic systems measured by the CO_2_-TPD method, high BET surface area, and crystallite size of CaO phase. Meanwhile, the highest FAME yield (98.5%) was achieved in the reaction performed at 220 °C on 10% SrO/BEA (Si/Al = 300) system, but with lower TG conversion (87.8%). Such good catalytic behavior of alkaline earth metal oxide-zeolite BEA systems was confirmed by the methanol sorption investigation on the surface of the catalysts. Especially, the best sorption properties were shown precisely by the catalysts with CaO and SrO loading. The results of the catalytic activity confirmed that alkaline earth metal oxide-zeolite BEA catalysts ensure high biodiesel efficiency in the transesterification reaction. The results of catalytic tests prove that CaO and SrO catalysts can be potential catalysts for use in the production of biodiesel on an industrial scale.

## Figures and Tables

**Table 1:** The results of the transesterification process performed over various alkaline earth metal oxide catalysts calcined in an air atmosphere for 4 h at 500 °C (10% MgO/BEA, 10% CaO/BEA) or 600 °C (10% SrO/BEA).

Catalyst	Reaction Temperature [°C]	Reaction Time [h]	Molar Ratio Methanol/Oil	Calcination Temperature [°C]	Catalyst Weight [g]	Triglycerides Conversion [%]	FAME Yield [%]
BEA (Si/Al = 25)	180	2	9:1	500	0.5	33.9	3.0
	220					68.7	30.0
BEA (Si/Al = 300)	180	2	9:1	500	0.5	17.5	3.1
	220					52.5	25.0
10% MgO/BEA (Si/Al = 25)	180	2	9:1	500	0.5	66.8	46.0
	220					69.5	55.7
10% MgO/BEA (Si/Al = 300)	180	2	9:1	500	0.5	63.0	29.0
	220					59.7	74.4
10% CaO/BEA (Si/Al = 25)	180	2	9:1	500	0.5	90.4	78.2
	220					90.2	92.6
10% CaO/BEA (Si/Al = 300)	180	2	9:1	500	0.5	84.2	64.0
	220					90.5	94.6
10% SrO/BEA (Si/Al = 25)	180	2	9:1	600	0.5	80.0	74.8
	220					74.4	89.6
10% SrO/BEA (Si/Al = 300)	180	2	9:1	600	0.5	71.4	65.4
	220					87.8	98.5

**Table 2:** The specific surface area, pore volume, and average pore radius for calcined alkaline earth metal oxide catalysts supported on BEA zeolite.

Catalyst	BET Surface Area (m^2^/g)	t-Plot Micropore Area (m^2^/g)	t-Plot External Surface Area (m^2^/g)	Pore Volume (cm^3^/g)	t-Plot Micropore Volume (cm^3^/g)	Average Pore Size (nm)
10% MgO/BEA (Si/Al = 25)	305	191	113	0.576	0.098	9.85
10% MgO/BEA (Si/Al = 300)	314	253	62	0.083	0.130	3.28
10% CaO/BEA (Si/Al = 25)	346	227	119	0.557	0.117	8.99
10% CaO/BEA (Si/Al = 300)	326	262	64	0.091	0.135	3.23
10% SrO/BEA (Si/Al = 25)	401	249	152	0.681	0.129	8.27
10% SrO/BEA (Si/Al = 300)	404	289	115	0.114	0.149	2.45

**Table 3:** Crystallite size of the active phase of alkaline earth metal oxide catalysts supported on BEA zeolite, calculated from XRD data using the Scherrer equation.

Catalyst	Average Size of Oxide Crystallites [nm]
10% MgO/BEA (Si/Al = 25)	MgO = 12
10% MgO/BEA (Si/Al = 300)	MgO = 9
10% CaO/BEA (Si/Al = 25)	CaO = 16
10% CaO/BEA (Si/Al = 300)	CaO = 14
10% SrO/BEA (Si/Al = 25)	SrO = 9
10% SrO/BEA (Si/Al = 300)	SrO = 13

**Table 4:** The amount of CO_2_ adsorbed on the support surface (calcined in an air atmosphere for 4 h at 500 °C), alkaline earth metal oxide catalysts supported on BEA zeolites with a different silicon/aluminum ratio, calculated from the CO_2_-TPD profiles.

Catalytic Systems	Weak Centers [mmol/g] 100–300 °C	Medium Centers [mmol/g] 300–450 ° C	Strong Centers [mmol/g] 450–600 ° C	Total Basicity [mmol/g] 100–600 ° C
10% MgO/BEA (Si/Al = 25)	0.10	0.28	0.64	1.03
10% MgO/BEA (Si/Al = 300)	0.55	0.49	0.61	1.65
10% CaO/BEA (Si/Al = 25)	0.24	0.41	0.58	1.22
10% CaO/BEA (Si/Al = 300)	0.57	0.69	0.67	1.93
10% SrO/BEA (Si/Al = 25)	0.15	0.38	0.57	1.10
10% SrO/BEA (Si/Al = 300)	0.21	0.37	0.40	0.99

**Table 5:** Phase gradient used in the HPLC measurements.

Mobile Phase Gradient			Flow Rate [mL∙min^−1^]
Time [min]	Solvent A (%)	Solvent B (%)	
0	100	0	0.9
20	100	0	0.9
45	0	100	0.9
70	0	100	0.9
75	100	0	0.9

Solvent A: methanol; Solvent B: 2-propanol/hexane = 4/5; Injection Volume: 1 μL; Column Temperature: 25 °C.

## Data Availability

Data is contained within the article.
